# Indigenous‐Led Analysis of Important Subsistence Species Response to Resource Extraction

**DOI:** 10.1002/ece3.71170

**Published:** 2025-03-28

**Authors:** Kathleen A. Carroll, Fabian Grey, Nicholas Anderson, Nelson Anderson, Jason T. Fisher

**Affiliations:** ^1^ Quest Lab, Department of Natural Resources Science University of Rhode Island Kingston Rhode Island USA; ^2^ Whitefish Lake First Nation #459 General Delivery Atikameg Alberta Canada; ^3^ School of Environmental Studies University of Victoria Victoria British Columbia Canada

**Keywords:** coproduction, country food, moose, resource extraction, subsistence hunting, traditional ecological knowledge

## Abstract

Subsistence hunting, or “country food,” on traditional territories is essential for numerous Indigenous Peoples who face food insecurity. For many First Nations of Canada, subsistence hunting is also inextricably linked to traditional conservation practices, as hunting is an important way of engaging with nature. In Canada's boreal forest, large game such as moose ( *Alces alces* ) is a primary source of protein. However, resource extraction—including forestry and oil and gas—has shifted large game distributions and affected the availability and abundance of food resources. Here, the Indigenous authors designed the study and processed remote camera trap data, then sought out Western scientists to generate generalized linear models to evaluate moose habitat use and spatial‐numerical responses to possible stressors in north‐central Alberta, including fire, harvest, oil and gas extraction, and other disturbances. Together, through the coproduction of knowledge, we examined the effects of human‐caused stressors on moose habitat use by sex and age class. The proportion of various land cover types and human land use for resource extraction was important in moose habitat use. Notably, male, female, and young moose all used habitat differently and at different spatial scales. However, young moose (with their mothers) strongly selected natural forest disturbances such as burned areas but avoided human‐created disturbances such as petroleum exploration “seismic” lines. Female moose with young attempts to maximize forage opportunities do not use human‐disturbed forests in the same ways they use naturally disturbed areas. Our findings, in the context of Indigenous interpretation from remote cameras and community insights, have linked human disturbance to declines in moose densities and displacement from traditional hunting grounds. Evaluating and predicting shifts in large game distributions is critical to supporting Indigenous food security and sovereignty and identifying where industries operating on First Nations lands can better engage responsibly with First Nations.

## Graphical abstract

Subsistence hunting, or “country food,” is essential for Indigenous peoples who face high food insecurity and is critical for Indigenous Food Sovereignty. In the boreal of Canada, large game such as moose ( *Alces alces* ) are a primary source of protein for many First Nations, but resource extraction, including forestry practices and oil and gas extraction, has shifted large game distributions. We found that female moose with young do not use human‐disturbed forests in the same ways they use naturally disturbed areas, which aligns with observations from Indigenous communities and links human disturbance to declines in moose densities and displacement from traditional hunting grounds.

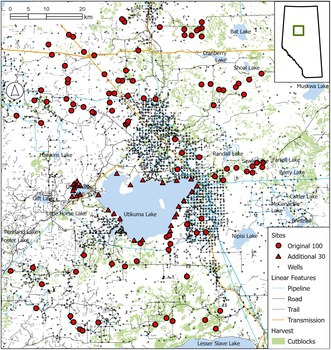

## 1 Introduction

Indigenous communities globally have relied on subsistence hunting of local species since time immemorial. However, communities most reliant on local ecosystem services, such as subsistence hunting, are the most vulnerable to threats associated with biodiversity and species loss (Díaz et al. 2006). Subsistence hunting sometimes exacerbates the deleterious effects of human resource use on local systems and species (Luz et al. 2017), particularly in regions where hunting is non‐selective or species are already at high risk due to habitat loss, climate change, or pollution (Lindsey et al. 2013; Ripple et al. 2016; Theriault 2011). However, the assumption that subsistence hunters solely maximize harvest in the short term rather than balancing foraging with conservation (i.e., considering the long‐term benefits of sustainability) is incorrect (Alvard 1994; Bodmer et al. 2020; VanStone 1974).

In North America, subsistence hunting (also referred to as “country food”) is closely linked with Indigenous conservation practices (Feit 1973; Gottesfeld 1994). These conservation practices range from limits on the number of individuals harvested to seasonal rotations of hunting grounds. In many parts of North America, ethical subsistence hunting (as determined by local Indigenous communities) is essential for food security (i.e., reliable access to nutritious and affordable food; Theriault 2011), supports Indigenous Food Sovereignty (the right to define and control food systems; Cidro et al. 2015; Armstrong 2020), and has additional social, cultural, and spiritual importance (Van Oostdam et al. 2005). Across much of North America, Indigenous harvesting is declining despite the importance of subsistence hunting for Indigenous communities (Gilbert et al. 2021; Shafiee et al. 2022), in part due to cost (i.e., permits, and equipment) and concerns about environmental contaminants in hunted food (Skinner et al. 2013), including cadmium, lead, arsenic, mercury, methylmercury, and other persistent organic pollutants (Chan et al. 2021). Reduced availability is another major reason, and many North American Indigenous communities have expressed resignation at the continued loss of their subsistence landbase (Westman and Joly 2019).

Industrial resource extraction has resulted in rapid changes in the densities, distributions, and communities of traditionally hunted species across Canada. For example, energy development, specifically oil and gas extraction, is one of the primary causes of the decline of woodland caribou (*Rangifer tarandus; atihk* in Cree) across western Canada (Hebblewhite 2017). Some species, such as wolves and bears, benefit from and consistently use anthropogenically created landscape features in Western Canada (Dickie et al. 2020, 2017), increasing these predators' hunting efficiency (McKenzie et al. 2012). However, not all species benefit from these industrial features, and many more actively avoid them (Fisher and Burton 2018). Increases in predator population size due to landscape development (Latham et al. 2011) and the high prey‐kill rates by such species associated with anthropogenic features (Boucher et al. 2022) impact traditionally hunted species across Canada, leading to additional pressures on country food.

Moose (*Alces alces; moswa in Cree*) are an important, but declining, subsistence resource for many First Nations of Canada (Kuzyk et al. 2018; Natcher et al. 2021; Priadka et al. 2022; Ross and Mason 2020) and there is widespread recognition that resource extraction impacts moose population dynamics, distributions, and predation rates in Alberta (Lamy and Finnegan 2019; Neilson and Boutin 2017). In the boreal, moose select for habitat that provides security when predator abundance is high (Ethier et al. 2024), and have lower occurrence in areas with pipelines, seismic lines, 3D seismic lines, unpaved roads, and new cut blocks (Dickie et al. 2022; Finnegan et al. 2023; Fisher et al. 2021; McKay and Finnegan 2023, 2022), which are often used by predators. This suggests that perceived predation risk is a strong driver of habitat selection, especially in areas with high human use. There are some potential benefits of human land use for moose, as forest cut blocks offer increased moose forage (Francis et al. 2021; Johnson and Rea 2023). However, the effects of herbicide treatment and predation risks in these cut blocks might outweigh the benefits, as moose in high herbicide‐use areas consume fewer forbs (Koetke et al. 2023). Finally, resource roads and trails associated with forestry and petroleum open access to previously remote areas, facilitating poaching, as has been observed by First Nations communities.

One of the Indigenous communities living in the western Nearctic boreal region is the Whitefish Lake First Nation (WLFN; including authors of this work), a Cree people who have noted the decline of moose across their territory. These observations have been conveyed to the provincial government and industry, who have demanded Western science as “proof” of these changes. Therefore, the WLFN led a Western‐science research project, conceived and executed by the community and informed by their traditional knowledge, current observations, and experience. This kind of Indigenous‐led research (see *Positionality Statement*) is rare—western science‐led community‐engaged research or coproduced research being more common. The community sought to better understand from both Indigenous and Western perspectives the degree to which moose abundance and habitat use have shifted across traditional territories with increasing human land use and the mechanisms for these changes in traditional hunting areas.

We sought to quantify the effects of human disturbance, fire, and land cover on moose relative abundance and spatial distribution using remote camera data to inform WLFN and other communities across Western Canada's subsistence hunting. Specifically, our goal was to determine the relative impacts of forest harvest, linear features (e.g., roads and pipelines), oil and gas extraction sites, forest cover types, and age of burned areas on moose distribution. We also sought to compare the relative effects of these features by age and sex, as male and female moose (with and without young) may select different features at different scales when balancing predation risk with forage availability. We generally expected moose with higher nutritional needs (e.g., females with young) might use a “riskier” (open) habitat when high‐quality forage is available and that all moose would be strongly associated with aquatic features due to their dietary needs (Fraser et al. 1984). We also expected differences in habitat use by sex and age, primarily between young moose (young of the year and young of last year) and males due to different dietary and safety needs. We also expected that the spatial scales at which landscape features explain moose distribution (Holland and Yang 2016) might differ by sex and age, as male moose may use larger areas, balancing foraging and seeking mates. In contrast, cows with young moose would likely be driven by the need for local high‐quality forage to support nursing young and offspring growth.

## 2 Methods

### 2.1 Study Area

**FIGURE 1:**
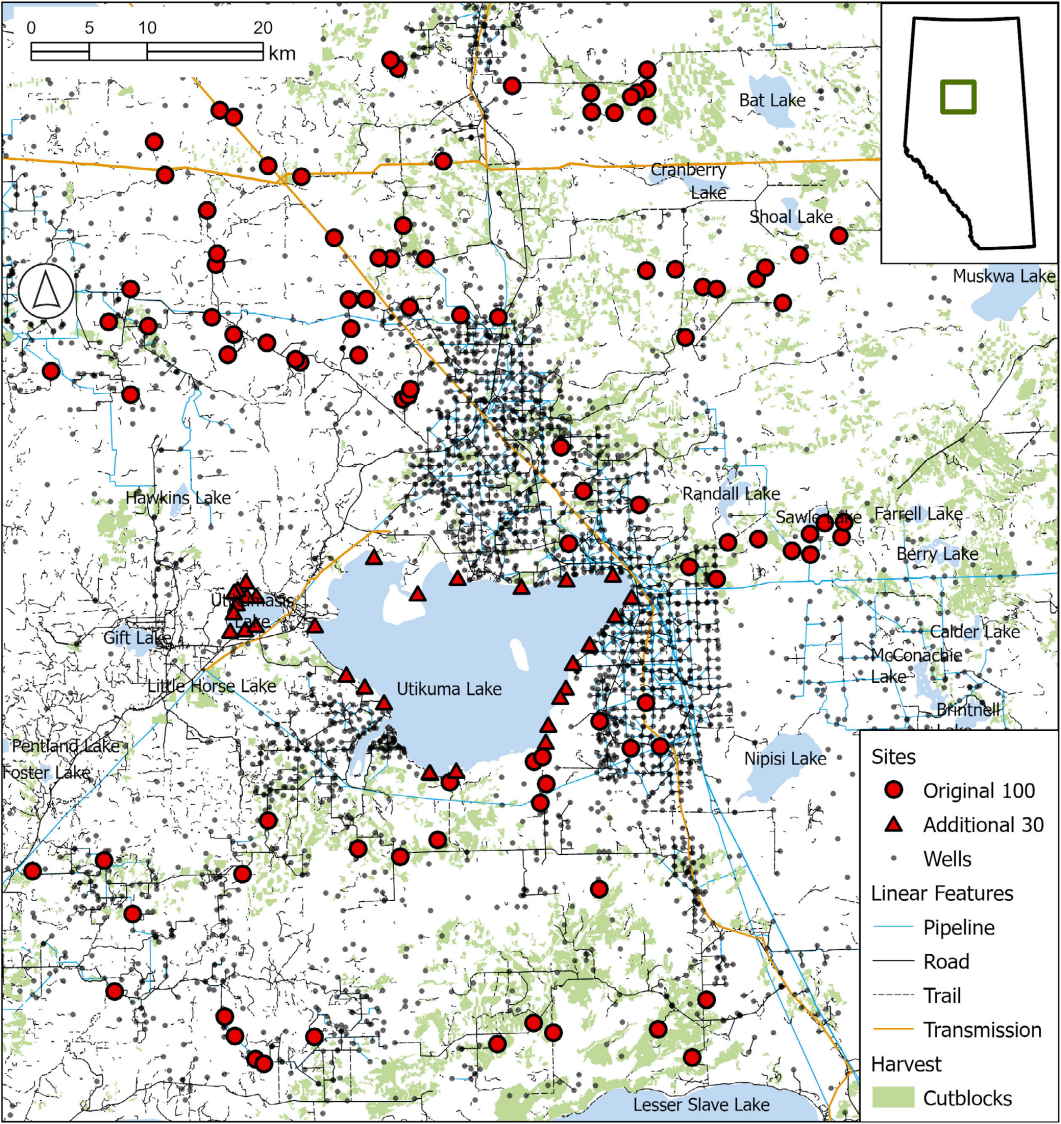
The study area shows camera locations, well sites, and other human disturbances.

Our study area encompassed the Whitefish Lake First Nation (WLFN) traditional territory, a Treaty 8 Territory (Fumoleau 2004) in north‐central Alberta, Canada (Figure 1), characterized by expansive central mixed‐wood forests interspersed with many small lakes, bogs, wetlands, fescue grasslands, both open and closed conifer stands, and closed shrublands (AMBI 2020). The Cree (including WLFN) have been living on this land for millennia, relying on its resources to survive in this cold and relatively nutrient‐poor boreal landscape. Recent industrial modification in the form of forestry and oil and gas extraction—which we refer to as “anthropogenic landscape features” and “anthropogenic disturbance”—is abundant across the landscape (Figure 1) and differs vastly from Indigenous landscape stewardship techniques. The area has experienced industrial forest harvest for a few decades. Harvested conifer stands are typically replanted and treated with glyphosate (N‐(phosphonomethyl) glycine) via helicopter, resulting in notable changes to plant communities and resources used by the Nation (Carroll et al. 2025). Widespread petroleum extraction is a more recent and even more widespread and diverse disturbance (Pickell et al. 2013; Pickell et al. 2015). Community Elders noted to the WLFN coauthors that the drastic human‐induced landscape changes on their Territory have resulted in precipitous declines of many important mammal and plant species. They also note that, like many other parts of Canada, increasing fire frequency is ‘extreme’ (sensu Gaboriau et al. 2022), with new burn records being set in recent years (CIFFC 2024; Canadian National Fire Database 2023; but see Chavardès et al. 2022).

### 2.2 Camera Trapping

WLFN coauthors conceived, designed, executed, and processed data for the research study. Sampling sites were assigned based on a constrained random stratified design, with advice from all coauthors, but WLFN determined final camera locations and conducted all fieldwork. The landscape was divided into four strata based on dominant canopy cover and hydrological conditions, and sites were randomly selected from these strata, with some constraints based on the logistics of access. Community members deployed 130 Reconyx Hyperfire 2 (Holmen, WI, USA) cameras between 2018 and 2023 (Figure 1). Of these cameras, 75 were deployed and active from December 2018 to April/May 2019, 25 more were added in March, and all 100 were active between March and November 2019. WLFN deployed an additional 30 cameras, which were active from June 2022 to July 2023. Cameras were placed ca. 1.5 m above ground at sampling sites. Sampling sites were active wildlife trails, and camera sensors were set to “high sensitivity” to record one image with each heat‐in‐motion detection, with no programmed delays between photographs, adopting techniques used in Fisher and Burton (2018). WLFN community members classified images into species using TimeLapse2 Image Analysis software (Greenberg et al. 2019). Of the deployed cameras, timelapse data and images were retrievable from 121 cameras (96 of the original 100 and 21 of the subsequent 30). There were no conflicts with cameras being damaged or removed; data loss was due to programming or other issues. Images were grouped across sampling periods for our analysis to ensure naïve occupancy was sufficiently large for meaningful results. Images were also categorized as male, female, young of the year (YOY), or young of last year (YLY) whenever possible.

### 2.3 Predictor Variables

We identified 30 predictor variables that were either previously linked to moose ecology or of interest to WLFN (Table 1). These variables fell into three broad categories: land cover, human features (or footprint), and burn area. Various land cover and human feature categories were combined based on their similar impacts (e.g., oil wells, gas wells, and other types of wells were all binned under “well”) or structures/functions (e.g., bogs and wetlands). We *z*‐scaled all predictors (mean = 0, SD = 1) before checking for spatial autocorrelation (Zuur et al. 2010). We then calculated the area covered by each predictor within different buffer sizes surrounding camera locations from 250 to 5000 m radii, sensu Fisher et al. (2011).

**TABLE 1:** Predictor variables used in model development for moose camera data.

Category	Variable	Description	Source
Land Cover	Exposed	Exposed soil	Alberta Satellite Land Cover (ASLC); Alberta Agriculture and Forestry, Government of Alberta
	Closed deciduous & mixed forest	Closed aspen/balsam poplar/birch and closed mixed wood	
	Grassland	Fescue grassland	
	Water	All water bodies	
	Bogs and wetlands	Graminoid wetlands, shrubby wetlands, undifferentiated wetlands, and black spruce bogs	
	Open conifer forest	Open undifferentiated coniferous forests	
	Closed shrubland	Closed upland shrub	
	Closed conifer forest	Close pine, closed Engelmann/white spruce and closed undifferentiated conifers	
Human Features	Transmission lines	Cleared corridors designated for the location of power transmission line infrastructure.	ABMI wall‐to‐wall human footprint inventory (2021)
	Borrow pits	Excavation outside of the road right‐of‐way is made solely to remove or provide borrowed material for the sub‐base construction for a specific roadway project. It includes any other associated infrastructure, such as access roads.	
	Clearing	Human footprint features related to various industrial activities.	
	Cultivation	Lands where the forest and/or shrubs have been removed to plant crops or grass species for livestock grazing.	
	Facilities	Human footprint features related to various industrial activities.	
	Mines	Human footprint features directly related to mining activities.	
	Trails	Cleared corridors surfaced with dirt or low vegetation for human/vehicle access.	
	Vegetated edges	Disturbed vegetation alongside road edges, railway edges including ditches, and other industrial features.	
	Wells	Ground cleared for an oil/gas well pad where at least one well is currently active.	
	Harvest	Areas where forestry operations have occurred (clear‐cut, selective harvest, salvage logging, etc.).	
	Recreation	Human footprint related to vegetated facilities and recreation.	
	Residential	Residential developments with buildings for human inhabitance.	
	Seismic	Cleared corridors created during hydrocarbon exploration.	
	Seismic 3D	Cleared corridors created during hydrocarbon exploration.	
	Pipeline	A line of underground and overground pipes, of substantial length and capacity, is used to convey petrochemicals. The physical clearing contains underground and above‐ground high‐pressure pipelines.	
	Roads	Non‐vegetated, impermeable surfaces used for motorized vehicle or aircraft transportation or access.	
Fire	Area burned (0–5 years)	Area burned between 2019 and 2023.	Fire perimeter data Wildfire management branch, Government of Alberta
	Area burned (6–10 years)	Area burned between 2014 and 2018.	
	Area burned (11–15 years)	Area burned between 2009 and 2013.	
	Area burned (16–20 years)	Area burned between 2004 and 2008.	
	Area burned (21–25 years)	Area burned between 1999 and 2003.	
	Area burned (26–29 years)	Area burned between 1995 and 1998. The shorter interval was based on there being no 1994 burn area data.	

### 2.4 Habitat Model

To control for repeated captures of animals (e.g., a single moose being photographed repeatedly), we first binned moose detections daily, where each site was assigned a positive detection if any moose were captured that day or an absence if no moose were detected that day. We then binned data monthly to estimate moose site use, controlling for the number of days each camera was functional. The number of days moose were present and absent at each site was combined to generate the response variable. In this approach, we assumed that if a moose was not detected at a site within the years of sampling, we could reliably state it did not occur there—rather than assuming false absence as in an occupancy framework (MacKenzie et al. 2003).

Moose habitat use was examined using binomial family generalized linear models (GLM). Our predictor variables for each model included the 30 land cover, human features, and burn area variables detailed above. We summarize the area of each response variable by generating 20 buffers for each camera site, ranging from 250 to 5000 m in diameter at 250‐m intervals. Our GLM models were run across all sites using bidirectional stepAIC model selection (Zhang 2016). A top model and scale were then determined for all data and for each age or sex category examined. After determining a top model, we examined predictor variance inflation factors (VIFs; ensuring each was < 4), component plus residual plots (to check for missing polynomial relationships (Fox et al. 2012)), and residual versus leverage plots for top models. The estimate and standard error were then assessed for each variable in each model.

### 2.5 Habitat Use by Age and Sex

After running the overall model with all moose data, we ran separate models for male, female (including those with and without calves), and young moose (YOY and YLY). We initially planned to analyze YOY and YLY separately, but these were grouped based on data limitations. Each age or sex model followed the same framework, used the same buffer sizes, and included the same variables as the overall moose model. These models were assessed using the same methods as the overall model.

### 2.6 Indigenous Knowledge and Interpretation

The Indigenous coauthors gathered knowledge about the wildlife and the landscape, past and present, on the Whitefish Lake First Nation's territory from conversations with their family and friends, elders, and community members. This knowledge was then shared between the Nation coauthors and the Western science coauthors (see the *Discussion* section, *Moose and Whitefish Lake First Nation*). Thus, our structure and strategy for engaging in meaningful coproduction of knowledge diverge from top‐down, prescribed Western social science methods. The ethical requirements attached to those Western methods were designed to prevent the exploitation of Indigenous peoples by Western scientists, where the latter use the knowledge and expertise of the former without credit. However, our work is a rare instance where Indigenous peoples are the researchers. Through this new path towards the coproduction of knowledge and science—which should be designed by and executed by Indigenous researchers (i.e., Indigenous‐led research)—Indigenous peoples and IK further eliminate barriers and reduce the ongoing hierarchization of knowledge.

## 3 Results

### 3.1 *Moose* Detections

**FIGURE 2:**
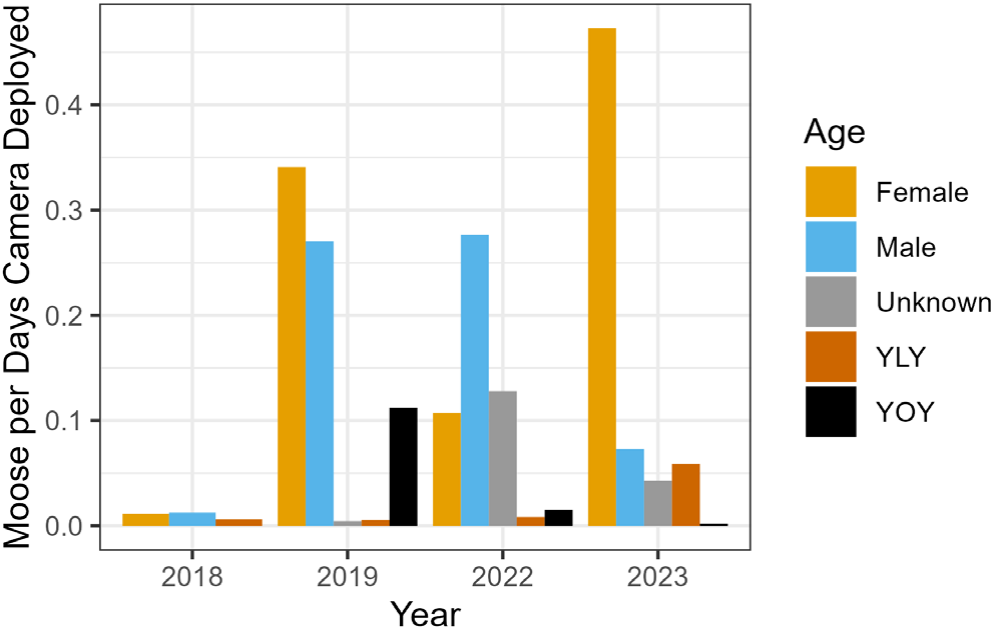
The count of adult male(s), adult female(s), adults of unknown sex, young of last year (YLY), and young of this year (YOY) identified each year from the camera array controlling for the number of trapping days cameras were deployed each year (total days for all cameras each year).

WLFN's cameras provided over 8000 moose images. When controlling for trapping days, the age and sex of moose detected varied across years (Figure 2). Female moose had the highest overall number of detections and were detected more often in 2019 and 2023 than males or young. Males had slightly more detections in 2018 and much higher detection rates than females in 2022. However, there were also many adults of unknown sex detected in 2022. The detections of young were highly variable across years, with many YOY detected in 2019, but none in 2018. Very few YLY were detected in any year, but 2023 had high detection rates of YLY.

### 3.2 Habitat Model

**FIGURE 3:**
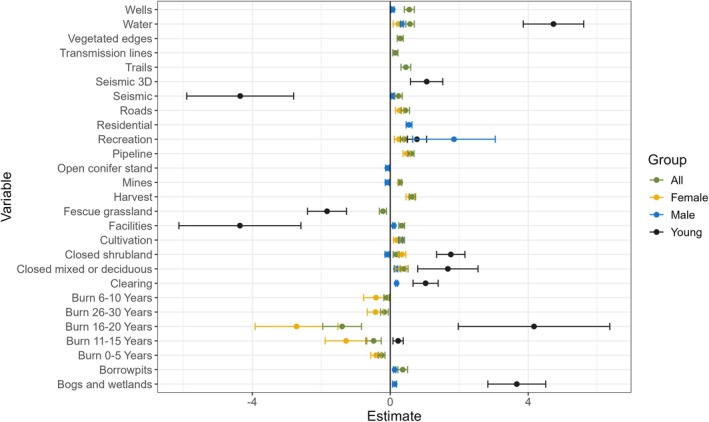
The effect size and direction of predictor variables on moose for all moose (including unknown adults; green) and male (blue), female (yellow), and young (black) moose separately. Error bars are based on GLM model standard errors.

**FIGURE 4:**
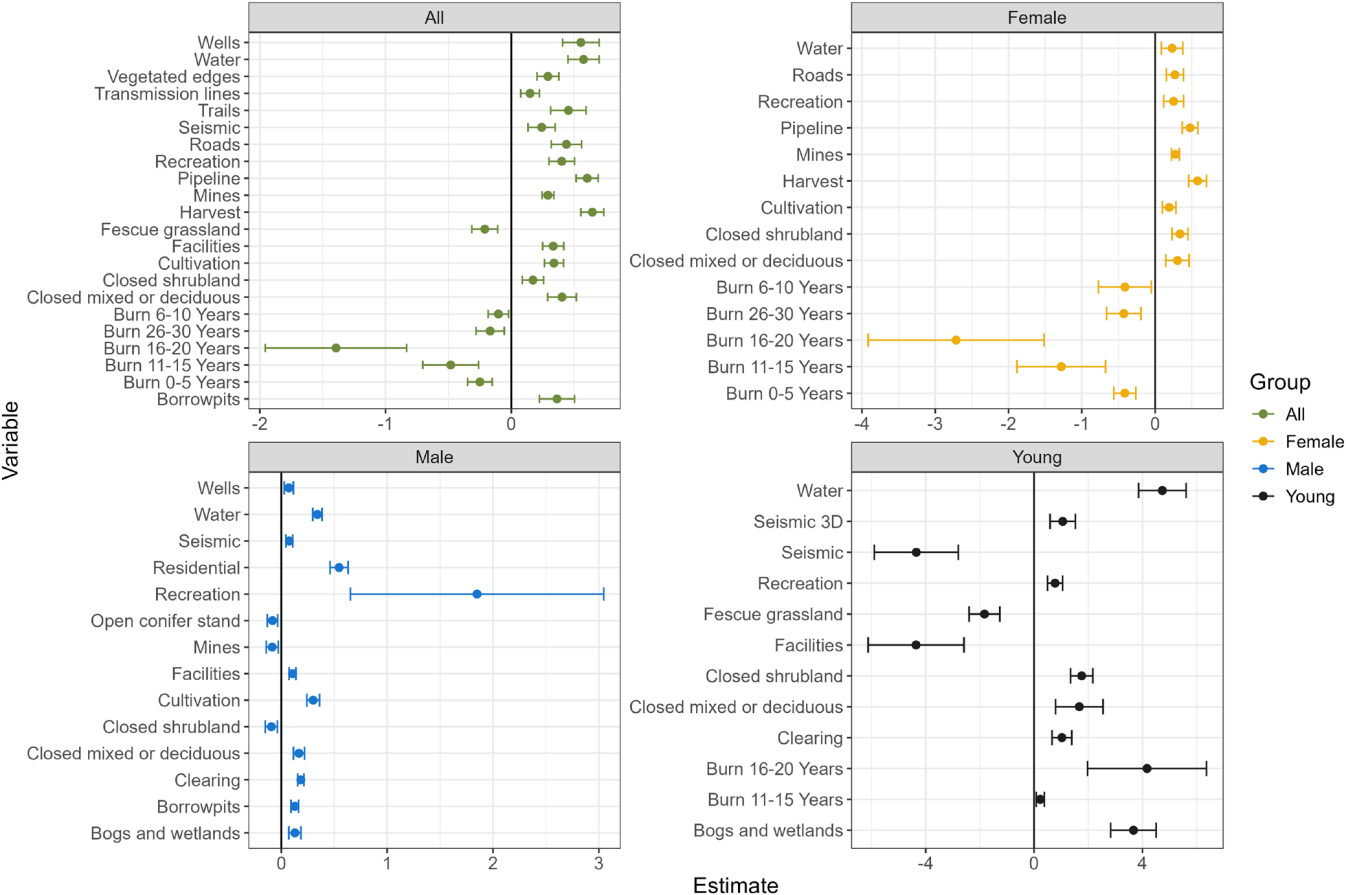
The effect size and direction of predictor variables with free axes on x for all moose (including unknown adults; green) and male (blue), female (yellow), and young (black) moose separately. Error bars are based on GLM model standard errors.

Overall moose habitat use (including male, female, unknown adult, and young) was best explained by 22 variables, 13 (nearly 60%) of which were human footprint features, 5 of which were related to the area burned, and the remaining 4 were land cover predictors (Figures 3 and 4). The area burned (including areas burnt 0–5, 6–10, 11–15, 16–20, and 26–30 years ago) and fescue grasslands had negative associations with moose habitat use. Thus, all human features, including harvest, pipeline, wells, trails, roads, recreation, borrow pits, cultivation, facilities, mines, vegetated road edges, seismic, and transmission lines, all had positive (albeit often small) β estimates.

### 3.3 Models by Sex and Age

Male, female, and young moose selected different habitat features at different scales. Male moose selected habitat at the broadest scale of the three groups, with a best‐fitting spatial scale of 4750‐m radius, followed by females at 2500‐m, and then young at 1500‐m radius. We did not distinguish between females with and without calves in the female group, as the habitat used by females with calves was captured in modeling young.

Nine of the fourteen predictors that best explained male moose habitat use were human footprint variables: recreation, residential, cultivation, clearings, borrow pits, facilities, mines, seismic lines, and wells (Figures 3 and 4). Male moose were the only group with no burn area predictors in the top model. Instead, the remaining five predictors were land cover types: water, closed mixed or deciduous forests, bogs and wetlands, closed shrublands, and open conifer stands. Of all predictors, recreation areas had the largest β estimate and wells had the smallest. Only closed shrublands, mines, and open conifer stands had negative associations with male moose habitat use, whereas all other variables had positive associations (Figures 3 and 4).

Female moose were the most detected (Figure 2) and selected habitat slightly differently than males (Figures 3 and 4). Area burned (including areas burnt 11–15 and 16–20 years ago) was the strongest predictor of female moose, with strong negative effect sizes. Female moose avoided nearly all burned areas but had positive habitat associations with harvest, pipelines, and many other human features. Unlike males, females had positive associations with closed shrubland and mines.

Young moose, which had the lowest detection rate (Figure 2), had the strongest effect sizes of model predictors and the largest number of predictors that differed from the other groups (Figures 3 and 4). Five of the twelve variables that predicted young moose habitat were human footprint variables. Young moose were the only group to have positive associations with burned areas (including areas burnt 11–15 and 16–20 years ago). Like female moose, young moose had positive associations with closed shrublands. However, there were two predictors not in the top female moose models where young moose differed from male moose, including facilities and seismic lines. Young moose strongly avoided both features, despite their very slight positive association with adult male moose use (Figures 3 and 4).

## 4 Discussion

Industrial resource extraction has altered moose relative abundance and distribution across the nearly 10,000 km^2^ of the Whitefish Lake First Nation territory, just as observed by elders and community members. Human footprint metrics explained variance in moose detections in all models, though often with very small effect sizes. However, the young moose model, where we expected to see strong selection for high‐quality forage areas in open spaces, had the strongest negative relationship with anthropogenic landscape features (Figures 3 and 4). The strong negative relationship between young moose and petroleum exploration “seismic” lines (Dabros et al. 2018) and industrial processing facilities (Fisher and Burton 2018) supports the observations of Indigenous community members and reiterates the importance of both broad and local‐scale impacts of human land use on boreal species important to Indigenous communities. Importantly, young moose strongly selected for slightly older burned areas, suggesting that early‐seral forage generated by natural disturbance is important, but that human‐caused disturbance (which also has ample early‐seral forage vegetation (Routh and Nielsen 2021)) is not equivalent to the young, open‐canopy patches created by fire. Fire (or here, burned area) has a large and important role in boreal environments but is rapidly shifting in size and severity across Canada, impacting wildlife species (DeMars et al. 2019; Palm et al. 2022). The roles of both fire and human land use as strong drivers in moose habitat selection are well known (DeMars et al. 2019; Dickie et al. 2020; Ethier et al. 2024; Fisher and Burton 2018; Fisher and Wilkinson 2005; Johnson and Rea 2023), but we highlight the important distinction between open areas caused by fire versus those caused by resource extraction for young moose seeking high‐quality forage. For traditional territories highly impacted by development, such as WLFN's territory, this has substantial consequences for food security and sovereignty.

### 4.1 Moose and Whitefish Lake First Nation

Empirical data show young moose strongly avoid seismic and facilities, which may in part explain WLFN community members' observations about declining moose populations in historic hunting grounds. Traditionally, WFLN has always hunted within the local area (ranging about 5–10 km) to harvest moose. Now, it takes 6 or 7 days of searching, and members must go further into the bush to harvest moose since the moose population is down and moose are using the landscape differently. Hunts taking longer and requiring further travel takes members away from their land, further eroding one of the key reasons members hunted. Extended travel times result in WLFN members spending large amounts of money on gas and food to go hunting and often must drive more than 5 hours. One of the consequences of this change, which WLFN is concerned about, is the loss of harvesting practices of the past. Traditionally, cows weren't harvested, but those practices are not followed these days because members must take what they can when the opportunity comes (harvest when seen). Members recognize that taking a cow takes out all its future offspring. Thus, WLFN members feel they must bear the responsibility of relearning how and where to hunt anew. Another consequence is the loss of cultural knowledge for younger generations. Elders note that the younger generation is choosing not to hunt because the changing moose availability changes how it is used as a staple. This results in younger members not only missing out on learning how to hunt but also missing out on other important knowledge like how to track moose and how to look for moose forage and other animal signs.

The cultural practice of hunting is not the only loss from changing moose habitat. Members of WLFN note that moose are a very important source of food and are deeply tied to the health of the land, water, and plants. They note that if the overall environment is healthy, so is the moose, and so is WLFN. WLFN members view eating moose regularly as critical for community members to get naturally occurring minerals since moose eat many medicinal plants. As they see a decline in important plants due to post‐harvest herbicide use, they recognize that lower‐quality medicinal plants result in lower‐quality moose. This issue is much broader than moose, though; warming lakes have resulted in more algae, removing plants that WLFN uses and moose eat. WLFN community members also have limited fishing due to mercury, and there are growing concerns over waterfowl health—all issues that contribute to less and less connection to the land and more reliance on Western staples.

### 4.2 Broader Implications for Indigenous Food Sovereignty and Security

Identifying how industrial land use impacts subsistence species is only one component of the much larger issues facing Indigenous communities and Indigenous Food Sovereignty and Security efforts (Batal et al. 2021). The rising costs of hunting and food contamination concern Indigenous communities reliant on country food (Chan et al. 2021; Shafiee et al. 2022). These issues are only exacerbated by species declines and changes in how species occupy and use landscapes as human pressures and fires increase. Concerns over access to high protein sources, specifically moose for WLFN, are evident. Community members note the challenges associated with finding moose, a lack of wildlife tracks, and evidence of browsing in harvested areas despite the possibility that regenerating stands result in increased forage (Koetke et al. 2023). Without access to subsistence species like moose, Indigenous Food Security is increasingly out of reach for Indigenous Peoples in these systems. Thus, it is critical for Indigenous Peoples to gain additional knowledge of how these human features are impacting species habitat use, which WLFN has done using surveys and remote cameras, and to be able to harvest moose when found. Supporting these efforts through the coproduction of research based on data collection and questions led by WLFN is one critical component of supporting Indigenous Food Sovereignty and Security.

More broadly, WLFN's concerns are reflected in the concerns of Indigenous communities across the boreal. Loss of cultural capital, the negative impacts of climate change, and the loss of overall community health and well‐being resulting from food insecurity are all broad issues that manifest in various ways across Indigenous communities in Canada (Priadka et al. 2022; Hirabayashi et al. 2022; Shafiee et al. 2022). Such concerns, like climate change, are exacerbated by development, and the impact of development in terrestrial systems (like in WLFN traditional territories) is mirrored in aquatic and marine systems (Jonasson et al. 2019; Islam and Berkes 2016). However, across Canada, governance and policy conflicts remain the biggest barriers to food security for Indigenous communities (Loring and Gerlach 2015). While Loring and Gerlach were correct that food security in Canada is primarily driven by socio‐political issues (2015), they noted that biogeography and ecology were less impactful. In the decade since their review, our results indicate Indigenous Food Sovereignty and Security are increasingly becoming both an ecological and biogeographical one as well.

### 4.3 The Importance of Coproduction of Research

The coproduction of science between Indigenous communities and Western scientists, based on clear expectations and relationships with knowledge‐holders (Adams et al. 2023; Huntington 2000), can provide insights and ecological understandings that might otherwise be missed and facilitate cultural continuity (Skroblin et al. 2021; Thompson et al. 2020). However, there are still many barriers to weaving together Indigenous and Western ways of knowing that can hamper collaborations (Smith 2021), such as biases that lead to a sense of the hierarchization of knowledge (Brook and McLachlan 2005). These barriers are slowly being surmounted as converging and diverging perspectives and values are addressed through Indigenous–Western science partnerships (Bélisle et al. 2022). Excitingly, there is growing recognition that Indigenous‐centered knowledge and Indigenous‐led research are essential for conservation on traditional territories and across ecosystems more broadly (Fisher et al. 2021; Rayne et al. 2020). However, much must be done to build relationships with knowledge‐holders and meaningful collaborations between those practicing Western science and Indigenous Peoples. Here, we have worked together to coproduce a research paper based on Indigenous‐led research and their knowledge of the land—and changes to wildlife communities—to add to a slowly growing body of like research. Finally, we urge industries operating on First Nations lands to better engage responsibly with First Nations and for Indigenous conservation and stewardship to be upheld in policy.

## Author Contributions

**Kathleen A. Carroll:** formal analysis (lead), methodology (equal), visualization (lead), writing – original draft (lead), writing – review and editing (equal). **Fabian Grey:** conceptualization (lead), data curation (lead), methodology (equal), resources (equal), writing – original draft (equal), writing – review and editing (equal). **Nicholas Anderson:** conceptualization (equal), data curation (equal), methodology (equal), writing – review and editing (equal). **Nelson Anderson:** conceptualization (equal), data curation (equal), methodology (equal), writing – review and editing (equal). **Jason T. Fisher:** conceptualization (supporting), data curation (supporting), funding acquisition (equal), project administration (equal), resources (equal), supervision (equal), writing – original draft (equal), writing – review and editing (equal).

## Conflicts of Interest

The authors declare no conflicts of interest.

## Positionality Statement

Our camera trap study was conceived by members of the Whitefish Lake First Nation (WLFN), and non‐Indigenous scientists (K.A.C., J.T.F.) provided expertise in data analysis and statistical interpretation. All authors were engaged throughout the research and study design. WLFN coauthors (F.G., N.A., & N.A.) consulted with Elders for their expertise and perspectives on moose ecology and changes in habitat use before and during the writing process.

## Data Availability

All relevant data are cited in the text or are the property of Whitefish Lake First Nation and other First Nations of Canada (see the CARE Principles for Indigenous Data Governance: https://www.gida‐global.org/care). Please contact Whitefish Lake First Nation coauthors with further questions regarding access to these datasets.
